# The genome sequence of the ferruginous bee-grabber, *Sicus ferrugineus *(Linnaeus, 1761)

**DOI:** 10.12688/wellcomeopenres.17748.1

**Published:** 2022-03-14

**Authors:** University of Oxford and Wytham Woods Genome Acquisition Lab, Darwin Tree of Life Barcoding collective, Wellcome Sanger Institute Tree of Life programme, Wellcome Sanger Institute Scientific Operations: DNA Pipelines collective, Tree of Life Core Informatics collective, Will Nash, Darwin Tree of Life Consortium

**Affiliations:** 1 Earlham Institute, Norwich, UK

**Keywords:** Sicus ferrugineus, ferruginous bee-grabber, genome sequence, chromosomal, Diptera

## Abstract

We present a genome assembly from an individual male *Sicus ferrugineus *(the ferruginous bee-grabber; Arthropoda; Insecta; Diptera; Conopidae). The genome sequence is 312 megabases in span. The majority of the assembly (99.67%) is scaffolded into 5 chromosomal pseudomolecules, with the X and Y sex chromosomes assembled. The complete mitochondrial genome was also assembled and is 16.9 kilobases in length.

## Species taxonomy

Eukaryota; Metazoa; Ecdysozoa; Arthropoda; Hexapoda; Insecta; Pterygota; Neoptera; Endopterygota; Diptera; Brachycera; Muscomorpha; Conopoidea; Conopidae; Myopinae; Sicus; *Sicus ferrugineus* (Linnaeus, 1761) (NCBI:txid1219204).

## Background

The ferruginous bee-grabber, *Sicus ferrugineus* (Linnaeus 1761), is the commonest and most abundant member of the dipteran family Conopidae (thick-headed flies) found in the British Isles (Conopid Recording Scheme of Britain & Ireland, personal communication). Widespread throughout Europe ( Stuke, 2017), these enigmatic flies inhabit grassland, woodland, hedgerow and garden habitats. Often seen resting on and around flowering plants, on which they feed, adults hold their elongated abdomens curled under the body ( Smith, 1969). At such food sources, *S. ferrugineus* females can readily be seen ‘grabbing’ several species of bumblebee ( *Bombus* spp.) both in the air and on surfaces ( Schmid-Hempel & Schmid-Hempel, 1996b). *S. ferrugineus* is an endoparasite of these bees, and these lunging ‘grabs’ are usually the point of egg delivery. Female *S. ferrugineus* bear specialised abdominal structures used to seize and inject a single egg into the body cavity of the target bee. These include the theca, a grasping structure under sternite 5. The theca is notably smaller in *S. ferrugineus* than that of the only other recorded British *Sicus* species, *S. abdominalis* ( Smith, 1969), and is the primary morphological structure used for identifying species of this genus. *S. ferrugineus* eggs bear a hooked micropyle ( Kotrba, 2011; Smith, 1969) and, once hatched, the resulting larva feeds on the haemolymph of the host, reaching pupal stage in around 11 days ( Schmid-Hempel & Schmid-Hempel, 1996a). The high-quality *S. ferrugineus* reference genome presented here is the first full genome sequence of a conopid fly and presents a unique opportunity to better understand the fascinating parasitic ecology of this species.

## Genome sequence report

**Figure 1.**
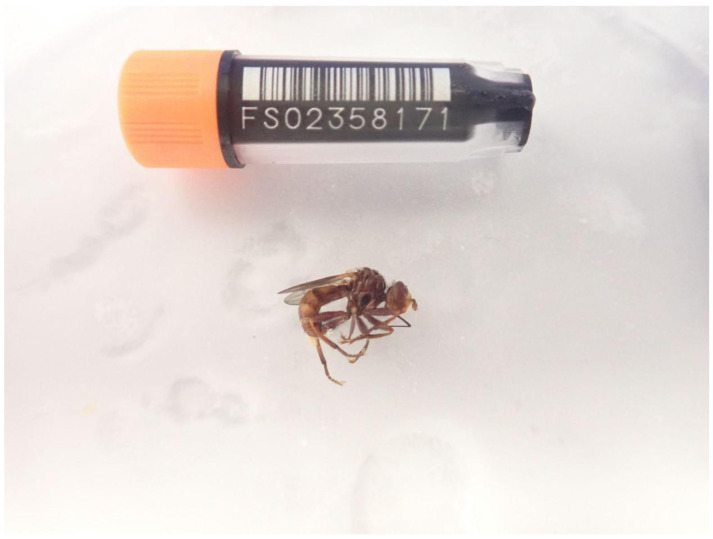
Image of the *Sicus ferrugineus* specimen taken prior to preservation and processing.

The genome was sequenced from a single male *S. ferrugineus* collected from Wytham Great Wood, Oxfordshire (Biological vice-county: Berkshire), UK (latitude 51.770, longitude -1.339) ( Figure 1). A total of 49-fold coverage in Pacific Biosciences single-molecule HiFi long reads and 83-fold coverage in 10X Genomics read clouds were generated. Primary assembly contigs were scaffolded with chromosome conformation Hi-C data. Manual assembly curation corrected 103 missing/misjoins, reducing the assembly size by 0.16% and the scaffold number by 65.88%, and increasing the scaffold N50 by 86.05%.

**Figure 2.**
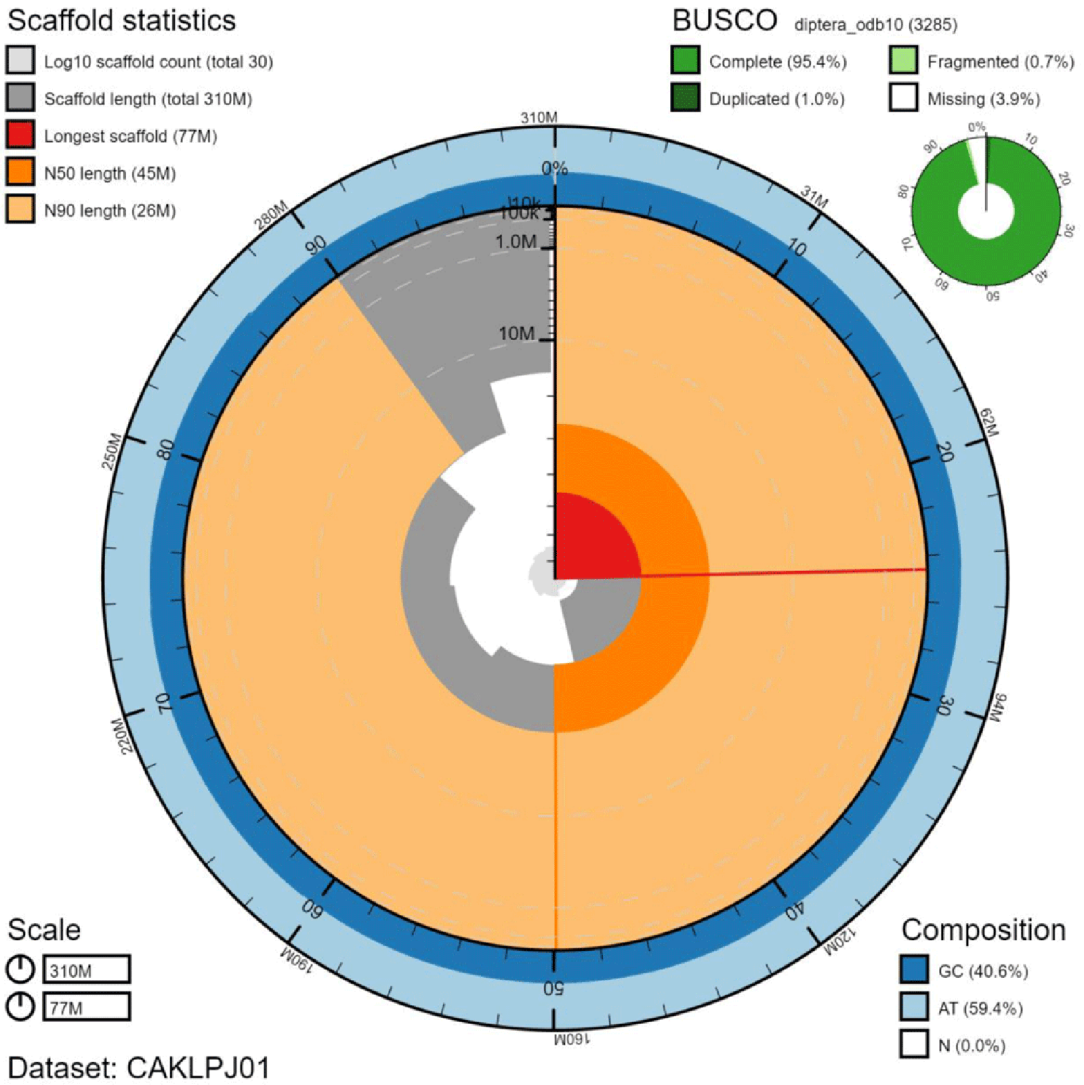
Genome assembly of *Sicus ferrugineus*, idSicFerr1.1: metrics. The BlobToolKit Snailplot shows N50 metrics and BUSCO gene completeness. The main plot is divided into 1,000 size-ordered bins around the circumference with each bin representing 0.1% of the 311,933,319 bp assembly. The distribution of chromosome lengths is shown in dark grey with the plot radius scaled to the longest chromosome present in the assembly (76,899,290 bp, shown in red). Orange and pale-orange arcs show the N50 and N90 chromosome lengths (44,927,291 and 26,327,110 bp), respectively. The pale grey spiral shows the cumulative chromosome count on a log scale with white scale lines showing successive orders of magnitude. The blue and pale-blue area around the outside of the plot shows the distribution of GC, AT and N percentages in the same bins as the inner plot. A summary of complete, fragmented, duplicated and missing BUSCO genes in the diptera_odb10 set is shown in the top right. An interactive version of this figure is available at https://blobtoolkit.genomehubs.org/view/idSicFerr1.1/dataset/CAKLPJ01/snail.

The final assembly has a total length of 312 Mb in 29 sequence scaffolds with a scaffold N50 of 44.9 Mb ( Table 1). The majority, 99.67%, of the assembly sequence was assigned to 7 chromosomal-level scaffolds, representing 5 autosomes (numbered by sequence length), and the X and Y sex chromosomes ( Figure 2– Figure 5; Table 2). Orientation and location of some pieces of heterochromatic repeat is less clear than for other regions of the assembly, particularly for heterochromatic regions in chromosome 2. The assembly contains many regions of pentameric repeat that cause problems with Hi-C mapping and are visible as drops in association.

**Table 1.** Genome data for *Sicus ferrugineus*, idSicFerr1.1.

*Project accession data*	
Assembly identifier	idSicFerr1.1
Species	*Sicus ferrugineus*
Specimen	idSicFerr1
NCBI taxonomy ID	1219204
BioProject	PRJEB48117
BioSample ID	SAMEA7520692
Isolate information	Male, thorax/abdomen (genome assembly), head (Hi-C)
***Raw data accessions***	
PacificBiosciences SEQUEL II	ERR7123980
10X Genomics Illumina	ERR7113581-ERR7113584
Hi-C Illumina	ERR7113578-ERR7113580
***Genome assembly***	
Assembly accession	GCA_922984085.1
*Accession of alternate* *haplotype*	GCA_922984205.1
Span (Mb)	312
Number of contigs	131
Contig N50 length (Mb)	14.3
Number of scaffolds	29
Scaffold N50 length (Mb)	44.9
Longest scaffold (Mb)	76.9
BUSCO * genome score	C:95.4%[S:94.3%,D:1.0%],F:0.7%,M:3.9%,n:3285

*BUSCO scores based on the diptera_odb10 BUSCO set using v5.1.2. C= complete [S= single copy, D=duplicated], F=fragmented, M=missing, n=number of orthologues in comparison. A full set of BUSCO scores is available at https://blobtoolkit.genomehubs.org/view/idSicFerr1.1/dataset/CAKLPJ01/busco.

**Figure 3.**
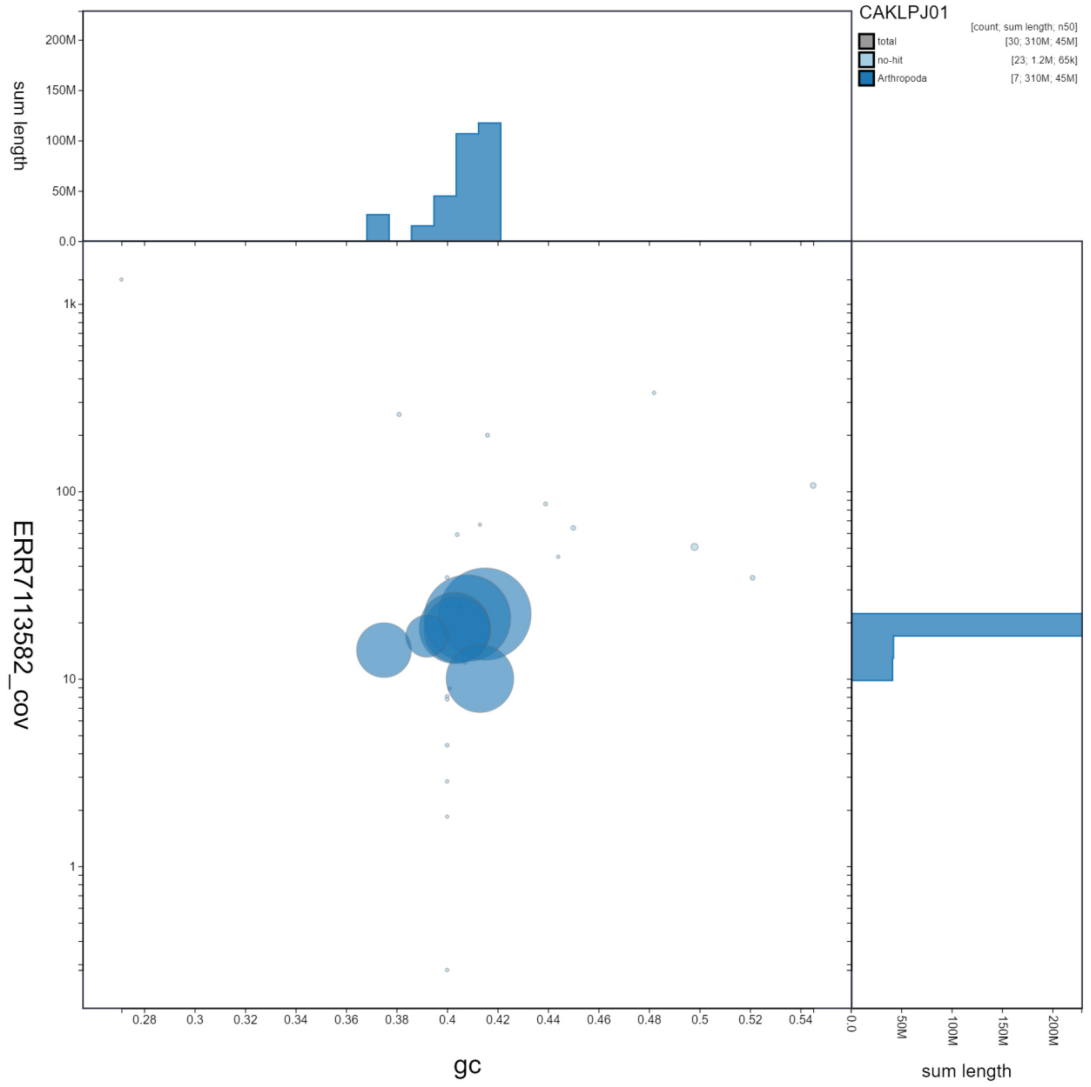
Genome assembly of *Sicus ferrugineus*, idSicFerr1.1: GC coverage. BlobToolKit GC-coverage plot. Scaffolds are coloured by phylum. Circles are sized in proportion to scaffold length. Histograms show the distribution of scaffold length sum along each axis. An interactive version of this figure is available at https://blobtoolkit.genomehubs.org/view/idSicFerr1.1/dataset/CAKLPJ01/blob.

**Figure 4.**
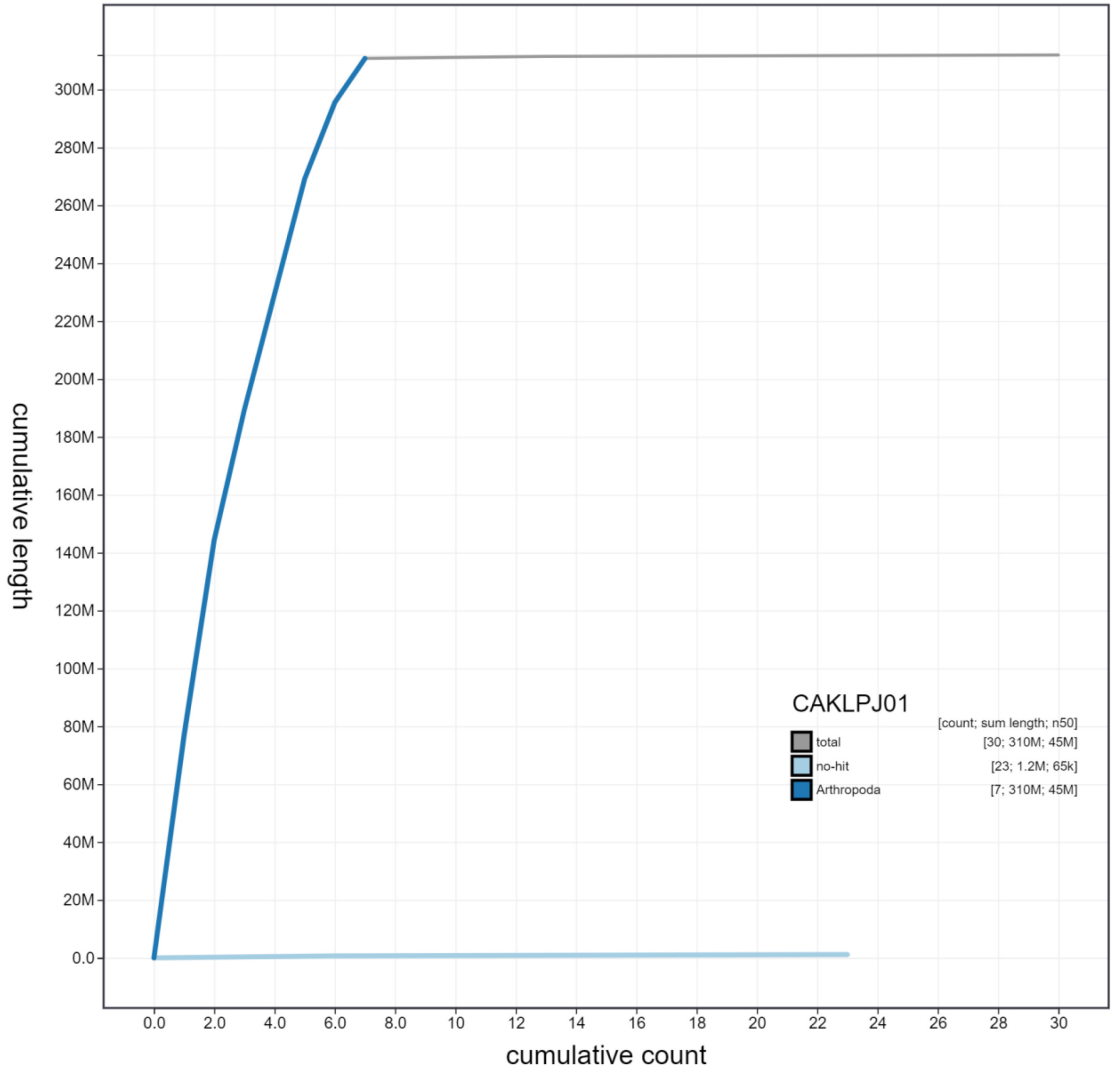
Genome assembly of *Sicus ferrugineus*, idSicFerr1.1: cumulative sequence. BlobToolKit cumulative sequence plot. The grey line shows cumulative length for all scaffolds. Coloured lines show cumulative lengths of scaffolds assigned to each phylum using the buscogenes taxrule. An interactive version of this figure is available at https://blobtoolkit.genomehubs.org/view/idSicFerr1.1/dataset/CAKLPJ01/cumulative.

**Figure 5.**
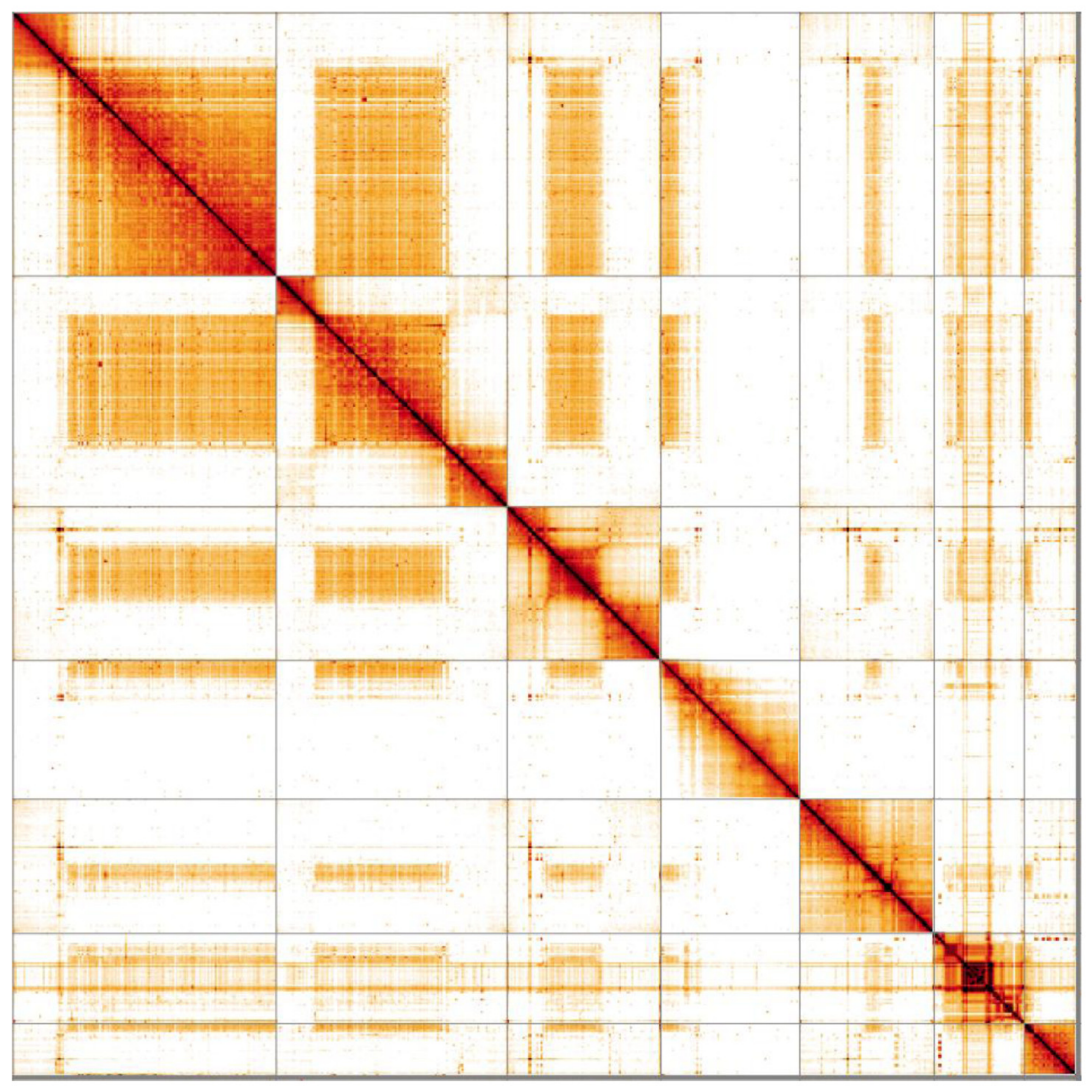
Genome assembly of *Sicus ferrugineus*, idSicFerr1.1: Hi-C contact map. Hi-C contact map of the idSicFerr1.1 assembly, visualised in HiGlass. Chromosomes are arranged in size order from left to right and top to bottom.

The assembly has a BUSCO v5.1.2 ( Manni *et al.,* 2021) completeness of 95.4% (single 94.3%, duplicated 1.0%) using the diptera_odb10 reference set (n=3285). While not fully phased, the assembly deposited is of one haplotype. Contigs corresponding to the second haplotype have also been deposited.

**Table 2.** Chromosomal pseudomolecules in the genome assembly of *Sicus ferrugineus*, idSicFerr1.1.

INSDC accession	Chromosome	Size (Mb)	GC%
OV277348.1	1	76.90	41.5
OV277349.1	2	67.51	40.8
OV277350.1	3	44.93	40.3
OV277352.1	4	39.27	40.4
OV277354.1	5	15.24	39.2
OV277351.1	X	40.60	41.3
OV277353.1	Y	26.33	37.5
OV277355.1	MT	0.02	27.1
-	Unplaced	1.14	45.3

## Methods

### Sample acquisition and DNA extraction

One male *S. ferrugineus* sample, idSicFerr1, was collected from Wytham Great Wood, Oxfordshire, (Biological vice-county: Berkshire), UK (latitude 51.770, longitude -1.339) by Liam Crowley, University of Oxford, on 15 June 2020. The specimen was caught in grassland with a net, identified by the same individual, snap-frozen on dry ice and stored using a CoolRack.

DNA was extracted at the Tree of Life laboratory, Wellcome Sanger Institute. The idSicFerr1 sample was weighed and dissected on dry ice with tissue set aside for Hi-C sequencing. thorax/abdomen tissue was cryogenically disrupted to a fine powder using a Covaris cryoPREP Automated Dry Pulveriser, receiving multiple impacts. Fragment size analysis of 0.01-0.5 ng of DNA was then performed using an Agilent FemtoPulse. High molecular weight (HMW) DNA was extracted using the Qiagen MagAttract HMW DNA extraction kit. Low molecular weight DNA was removed from a 200-ng aliquot of extracted DNA using 0.8X AMpure XP purification kit prior to 10X Chromium sequencing; a minimum of 50 ng DNA was submitted for 10X sequencing. HMW DNA was sheared into an average fragment size between 12–20 kb in a Megaruptor 3 system with speed setting 30. Sheared DNA was purified by solid-phase reversible immobilisation using AMPure PB beads with a 1.8X ratio of beads to sample to remove the shorter fragments and concentrate the DNA sample. The concentration of the sheared and purified DNA was assessed using a Nanodrop spectrophotometer and Qubit Fluorometer and Qubit dsDNA High Sensitivity Assay kit. Fragment size distribution was evaluated by running the sample on the FemtoPulse system.

### Sequencing

Pacific Biosciences HiFi circular consensus and 10X Genomics Chromium read cloud sequencing libraries were constructed according to the manufacturers’ instructions. Sequencing was performed by the Scientific Operations core at the Wellcome Sanger Institute on Pacific Biosciences SEQUEL II (HiFi) and Illumina HiSeq X (10X) instruments. Hi-C data were generated in the Tree of Life laboratory from head tissue of idSicFerr1 using the Arima v2 kit and sequenced on a HiSeq X instrument.

### Genome assembly

Assembly was carried out with Hifiasm ( Cheng *et al.,* 2021); haplotypic duplication was identified and removed with purge_dups ( Guan *et al.,* 2020). One round of polishing was performed by aligning 10X Genomics read data to the assembly with longranger align, calling variants with freebayes ( Garrison & Marth, 2012). The assembly was then scaffolded with Hi-C data ( Rao *et al.,* 2014) using SALSA2 ( Ghurye *et al.,* 2019). The assembly was checked for contamination as described previously ( Howe *et al.,* 2021). Manual curation was performed using HiGlass ( Kerpedjiev *et al.,* 2018) and Pretext. The mitochondrial genome was assembled using MitoHiFi ( Uliano-Silva *et al.,* 2021), which performs annotation using MitoFinder ( Allio *et al.,* 2020). The genome was analysed and BUSCO scores generated within the BlobToolKit environment ( Challis *et al.,* 2020). Table 3 contains a list of all software tool versions used, where appropriate.

**Table 3.** Software tools used.

Software tool	Version	Source
Hifiasm	0.15.3	Cheng *et al.,* 2021
purge_dups	1.2.3	Guan *et al.,* 2020
SALSA2	2.2	Ghurye *et al.,* 2019
longranger align	2.2.2	https://support.10xgenomics.com/ genome-exome/software/pipelines/ latest/advanced/other-pipelines
freebayes	1.3.1-17- gaa2ace8	Garrison & Marth, 2012
MitoHiFi	2.0	Uliano-Silva *et al.,* 2021
HiGlass	1.11.6	Kerpedjiev *et al.,* 2018
PretextView	0.2.x	https://github.com/wtsi-hpag/ PretextView
BlobToolKit	2.6.4	Challis *et al.,* 2020

### Ethics/compliance issues

The materials that have contributed to this genome note have been supplied by a Darwin Tree of Life Partner. The submission of materials by a Darwin Tree of Life Partner is subject to the Darwin Tree of Life Project Sampling Code of Practice. By agreeing with and signing up to the Sampling Code of Practice, the Darwin Tree of Life Partner agrees they will meet the legal and ethical requirements and standards set out within this document in respect of all samples acquired for, and supplied to, the Darwin Tree of Life Project. Each transfer of samples is further undertaken according to a Research Collaboration Agreement or Material Transfer Agreement entered into by the Darwin Tree of Life Partner, Genome Research Limited (operating as the Wellcome Sanger Institute), and in some circumstances other Darwin Tree of Life collaborators.

## Data availability

European Nucleotide Archive: Sicus ferrugineus (ferruginous bee-grabber). Accession number PRJEB48117; https://identifiers.org/ena.embl/PRJEB48117.

The genome sequence is released openly for reuse. The *S. ferrugineus* genome sequencing initiative is part of the Darwin Tree of Life (DToL) project. All raw sequence data and the assembly have been deposited in INSDC databases. The genome will be annotated and presented through the Ensembl pipeline at the European Bioinformatics Institute. Raw data and assembly accession identifiers are reported in Table 1.

## Author information

Members of the University of Oxford and Wytham Woods Genome Acquisition Lab are listed here: https://doi.org/10.5281/zenodo.5746938.

Members of the Darwin Tree of Life Barcoding collective are listed here: https://doi.org/10.5281/zenodo.5744972.

Members of the Wellcome Sanger Institute Tree of Life programme are listed here: https://doi.org/10.5281/zenodo.6125027.

Members of Wellcome Sanger Institute Scientific Operations: DNA Pipelines collective are listed here: https://doi.org/10.5281/zenodo.5746904.

Members of the Tree of Life Core Informatics collective are listed here: https://doi.org/10.5281/zenodo.6125046.

Members of the Darwin Tree of Life Consortium are listed here: https://doi.org/10.5281/zenodo.5638618.
