# Supplementary material for: The genome sequence of the ferruginous bee-grabber, Sicus ferrugineus (Linnaeus, 1761)
Source: Wellcome Open Res. 2022 Mar 14;7:91. [Version 1] doi: 10.12688/wellcomeopenres.17748.1 (PMC10282563; doi:10.12688/wellcomeopenres.17748.1)
Supplement: Peer review history

# Peer review: Reviewer response for version 1

**DOI:** 10.21956/wellcomeopenres.19642.r58063

**Published:** 2023-06-07

**Authors:** Nico Fuhrmann

**Affiliations:** 1 Biogeography, University of Trier, Trier, Rhineland-Palatinate, Germany

The article presents the first chromosome-level genome assembly for the ferruginous bee-grabber, *Sicus ferrugineus*. It is the first publicly available genome sequence of an abundant member of the dipteran family Conopidae. Extracted from a single male individual, the authors chose to sequence the genome on the PacBio SEQUEL II and Illumina HiSeq X. The initial assembly was done with Hifiasm and followed by short-read polishing. Using Hi-C data for the genome scaffolding reduced the scaffold numbers significantly. The resulting assembly consists of five autosomes, the X- and Y-chromosome and the mitochondrion of the species. The quality analyses of the assembly indicates large scaffolds and 95% found complete dipteran BUSCOs. This high-quality genome sequence is available on the nucleotide database ENA. A genome annotation is still missing. However, the authors mention that the genome will be annotated in the future and available through Ensembl.

 The authors utilize state-of-the-art methods and shows that high afford results in high-quality genomic sequences. The detailed laboratory performance description makes replication very easy. The authors do provide a list of the utilized software and version numbers (table 3), which is important and helpful information. Publishing the genome annotation alongside the genomic sequence would have been a great addition to the article.

 The last sentence of the background section hints towards the importance of the genomic sequence. The authors explain the species biology very well. *S. ferrugineus*’ bumblebee parasitism renders it an important target for biomonitoring and conservation affords of key pollinator species in the ecosystem. The article should directly mention application possibilities of the assembly.

 One more open question is about an unplaced fraction of about 1 Mb in size, about 0.3% of the total assembly. It is worth investigating where these fragments originate or belong. A possibility is using BLAST to identify potential contaminations (e.g. endosymbionts, parasites, human). Please add the information if these fragments are not part of the *S. ferrugineus* genome.

Are sufficient details of methods and materials provided to allow replication by others?

Yes

Is the rationale for creating the dataset(s) clearly described?

Partly

Are the datasets clearly presented in a useable and accessible format?

Yes

Are the protocols appropriate and is the work technically sound?

Yes

Reviewer Expertise:

Evolution and population genomics

I confirm that I have read this submission and believe that I have an appropriate level of expertise to confirm that it is of an acceptable scientific standard.

# Peer review: Reviewer response for version 1

**DOI:** 10.21956/wellcomeopenres.19642.r55183

**Published:** 2023-03-08

**Authors:** Ludvik M Gomulski

**Affiliations:** 1 University of Pavia, Pavia, Italy

The article describes the sequencing and assembly of the first full genome of a conopid fly, the ferruginous bee-grabber, *Sicus ferrugineus*. The species is an endoparasite of bees. The female grabs and injects an egg into a target bee and the resulting larva feeds on the haemolymph of its host. The description of the methods is detailed and clear and the resulting genome covering the 5 autosomes and the X and Y sex chromosomes appears to be of excellent quality. Both the genome sequence and the complete mitochondrial genome were assembled. The genome sequence has not been annotated.

 The species author is missing for *Sicus abdominalis*.

Are sufficient details of methods and materials provided to allow replication by others?

Yes

Is the rationale for creating the dataset(s) clearly described?

Yes

Are the datasets clearly presented in a useable and accessible format?

Yes

Are the protocols appropriate and is the work technically sound?

Yes

Reviewer Expertise:

Insect functional genetics

I confirm that I have read this submission and believe that I have an appropriate level of expertise to confirm that it is of an acceptable scientific standard.
